# Establishing *in vitro* and *in vivo* Co-culture Models of *Staphylococcus epidermidis* and *Enterococcus faecalis* to Evaluate the Effect of Topical Fluoroquinolone on Ocular Microbes

**DOI:** 10.3389/fmed.2021.670199

**Published:** 2021-12-20

**Authors:** Han Woo Kim, Jiyeun Kate Kim, Indal Park, Sang Joon Lee

**Affiliations:** ^1^Department of Microbiology, College of Medicine, Kosin University, Busan, South Korea; ^2^Department of Ophthalmology, College of Medicine, Kosin University, Busan, South Korea

**Keywords:** *Enterococcus*, *Staphylococcus*, fluoroquinolone, endophthalmitis, topical eye drops, co-culture model

## Abstract

**Purpose:** To establish *in vitro and in vivo* ocular co-culture models of *Staphylococcus epidermidis* and *Enterococcus faecalis* and to study how various concentrations of moxifloxacin affect the survival of these two endophthalmitis-causing bacteria.

**Methods:** Standard strains of *S. epidermidis* and *E. faecalis* were used. Color detection agar plates were employed to distinguish their colonies. To establish the *in vitro* and *in vivo* co-culture models, *S. epidermidis* and *E. faecalis* were co-cultivated at different ratios for various periods. For the *in vivo* model, various volumes and concentrations of either a mono-culture or co-culture were inoculated into the lower conjunctival sac of rabbits. Finally, the newly developed *in vitro* and *in vivo* co-culture models were subjected to the moxifloxacin treatment to access its effect on *S. epidermidis* and *E. faecalis*.

**Results:** When *S. epidermidis* and *E. faecalis* were cultured separately in tryptic soy broth, their growth peaked and plateaued at approximately 16 and 6 h, respectively. When they were co-cultured, the growth peak of *S. epidermidis* got delayed, whereas the growth peak of *E. faecalis* did not change. The number of *E. faecalis* was significantly higher in the co-culture than that in the mono-culture. Treatment with moxifloxacin in the *in vitro* co-culture model rapidly decreased the number of *S. epidermidis* cells at doses ≥ 0.125 μg/ml. In contrast, the number of *E. faecalis* did not change significantly up to 16 μg/ml moxifloxacin. In *in vivo* co-culture (at 1:1), the *S. epidermidis* count decreased in a pattern similar to that seen in *in vivo* mono-culture and was barely detectable at 24 h after inoculation. In contrast, the of *E. faecalis* count increased up to 16 h and then decreased. When moxifloxacin was applied (zero, one, or two times) to this model, the *S. epidermidis* count decreased in proportion to the number of treatments. In contrast, the *E. faecalis* count increased with moxifloxacin treatment.

**Conclusions:** The *in vitro* and *in vivo* co-culture models of *S. epidermidis* and *E. faecalis* were established to determine the influence of moxifloxacin eye drops on these bacteria. The results clearly show that the moxifloxacin eye drops can make *E. faecalis* dominant on the ocular surface.

## Introduction

Cataract surgery is one of the most common surgical procedures worldwide. Owing to advancements in surgical techniques and materials, cataract surgery is now faster and safer (1). Nonetheless, various complications, including posterior capsular tear, loss of endothelial cells, corneal stromal burn, retinal detachment, and postoperative endophthalmitis (PE), still occur (2). Acute PE is a rare prevalence 0.023–0.26% but devastating complication of cataract surgery (3–5).

PE is initiated by the entry of microorganisms into the intraocular space, and the type of invading microorganism is a critical determinant of visual outcomes (6, 7). Because most of the bacteria causing PE come from commensal conjunctival microbes (8), it is important to confirm the presence of certain bacteria on the ocular surface and to monitor changes in this bacterial community.

Recently, it has been reported that the distribution of the causative bacteria of postoperative PE changed in Korea, Sweden, and Taiwan (9–11). These changes may reflect alterations of conjunctival microflora in the PE patients. Nonetheless, it would be difficult to determine the changes in this ocular surface microbial community by human clinical trials because the incidence of endophthalmitis is too low to meet statistical power requirements of a study population. Therefore, we decided to investigate the changes in the causative bacteria of PE by constructing an experimental co-culture model using representative bacteria occurring on the ocular surface. *In vitro* and *in vivo* co-culture models based on various microbial species have enabled research into cell-cell interactions among eukaryotic and bacterial species and helped to mimic complex *in vivo* environment (12).

A healthy and balanced microbial community is essential for maintaining metabolic, physiological, and immunological functions. Commensal microorganisms of the ocular surface provide defense against competitors, by inhibiting the growth of more virulent bacterial strains (13). Nevertheless, a variety of factors, such as age, dry eye, contact lens use, immunocompromising diseases, medications, and environmental factors can shift commensal-microflora composition on the ocular surface (13–16). Such a shift may occur in response to increased use of topical antibiotics intended to prevent ocular infection. Previous studies reporting that the distribution of isolates causing PE is changing from coagulase-negative staphylococci (CNS) to enterococci suggest that external factors have altered the relationship between the two most common bacterial taxa in the conjunctiva (9–11).

Fluoroquinolones are bactericidal antibiotics that directly inhibit bacterial DNA synthesis (17). Topical fluoroquinolones are the prophylactic antibiotics most commonly used in ophthalmic surgery (18). Although no randomized clinical trials of surgical prophylaxis for controlling ocular infection have been conducted, topical fluoroquinolones are routinely employed perioperatively in eye clinics (19). The first three topical fluoroquinolones introduced as topical eye drops were 0.3% ciprofloxacin, 0.3% ofloxacin, and 0.3% norfloxacin. Subsequently, next generation fluoroquinolones, including 0.5% levofloxacin, 0.3% gatifloxacin, and 0.5% moxifloxacin, have been introduced. The most important attribute of a later generation fluoroquinolones is their enhanced activity against gram-positive bacteria relative to that of the previous generation of fluoroquinolones (17). Currently, a moxifloxacin solution, a fourth-generation fluoroquinolone, is the most popular topical eye antibiotic for ophthalmic surgery. Of note, the fluoroquinolones possess high antimicrobial activity against staphylococci, as confirmed in some studies; however, these drugs are known to have poor antimicrobial activity against *Enterococcus faecalis* (20–23). The selective antimicrobial activity of a fluoroquinolone may have an effect on the alteration of microbial community on the ocular surface and induce changes in causative isolates of PE from coagulase-negative staphylococci to enterococci.

At present, however the experimental studies have not been attempted to elucidate the mechanism underlying the changes in causative bacteria of PE. Here, we hypothesized that the major factor altering of causative isolates of PE is perioperative application of ophthalmic antibiotics such as moxifloxacin to the conjunctiva of patients (24). To test this hypothesis, we constructed *in vitro* and *in vivo* co-culture models by representative causative pathogens of PE: *S. epidermidis* and *E. faecalis*. Then, the models were subjected to a fluoroquinolone ophthalmic antibiotic, and responses of the two bacterial species to the antibiotic were examined.

## Methods

### Bacterial Strains and Growth Conditions

Standard strains of *E. faecalis* (ATCC29212) and *S. epidermidis* (KCTC1917; equivalent to ATCC12228) were purchased from the American Type Culture Collection and the Korean Collection of Type Cultures, respectively. Both strains were cultured either in tryptic soy broth (TSB) or on tryptic soy agar (TSA) at 37°C. These standard strains were used to set up the *in vitro* and *in vivo* co-culture models.

### *In vitro* Mono- and Co-cultures of *S. epidermidis* and *E. faecalis*

A single bacterial colony was streaked on a TSA plate and incubated for 24 h at 37°C. Then, colonies were collected and dispersed in TSB, and the suspension was diluted to optical density at 600 nm (OD_600_) of 0.1. The suspensions of the standard *S. epidermidis* or *E. faecalis* strains were prepared at equal concentrations (2.5 × 10^7^ CFU/mL) in 5 ml of TSB.

For the *in vitro* mono-culture model, each bacterial suspension was incubated for 24 h, and then 100 μl aliquots were collected at 2 h intervals for 12 and at 4 h intervals thereafter. The samples were diluted 1:10^5^ and 1:10^6^ with a 0.85% NaCl solution, and 100 μl of each diluted bacterial suspension was spread on a TSA plate. After incubation for 24 h, the colonies on the plates were counted by means of a colony counter.

For the *in vitro* co-culture model, the prepared bacterial suspensions of *S. epidermidis* and *E. faecalis* (2.5 × 10^7^ CFU/ml each) were mixed in equal volumes and co-cultured for 42 h. The mixture was cultivated in two media in parallel (TSB and PBS). Then, 100 μl of the bacterial suspension was collected at 2 h intervals for 16 h and at 4 h intervals thereafter. The samples were serially diluted, and 100 μl of the diluted suspension was spread on a color detection agar (CDA) plate (MB-C1611; MB-cell, Korea). On such a plate, colonies of *E. faecalis* and *S. epidermidis* can be distinguished by color: *E. faecalis* colonies are blue, whereas *S. epidermidis* colonies are white. After 24 h of incubation, the colonies of each bacterium were counted using the colony counter.

### Cultivation of *S. epidermidis* and *E. faecalis* With Various Concentrations of a Culture Supernatant

A single bacterial colony was streaked on a TSA plate and incubated for 24 h at 37°C. After that, colonies were collected and dispersed and diluted in TSB to an OD_600_ of 0.1. The bacterial suspension was cultivated in a shaking incubator (N-Biotek, South Korea). After cultivation for 24 h, a cell-free culture supernatant was prepared by centrifuging the culture media and passing the supernatant through a 0.2 μm syringe filter (Jet biofil, China). The culture supernatants of *E. faecalis* and *S. epidermidis* were next diluted to various concentrations (50, 80, and 100%) with TSB. To measure the effects of a culture supernatant on bacterial growth, 15 μl of the bacterial suspension (2.5 × 10^7^ CFU/mL) and 135 μl of each culture supernatant (separately) were mixed in a 96 well plate and incubated at 37°C with shaking. OD_600_ was measured on a microplate reader (Infinite M200; Tecan, USA) wih 30 min intervals for 24 h to build a growth curve.

### Analysis of the Impact of Moxifloxacin on *S. epidermidis* and *E. faecalis* in the *in vitro* Co-culture Models

*S. epidermidis* and *E. faecalis* suspensions were prepared at equal concentrations (2.5 × 10^7^ CFU/ml) in TSB and mixed at a ratio of either 1:1 or 95:5 (*S. epidermidis*: *E. faecalis*). The co-culture prepared at the ratio of 1:1 was incubated for 18 h, and 100 μl of the resultant suspension was withdrawn and mixed with 900 μl of TSB containing various concentrations of moxifloxacin (0–16 μg/ml; Tokyo Chemical Industry, Japan), and incubated for 14 h. Next, the suspension was serially diluted and plated on CDA. The co-culture prepared at the ratio of 95:5, *S. epidermidis* 95 ul and *E. faecalis* 5 ul, was mixed with TSB (900 ul) containing various concentrations of moxifloxacin and incubated for 18 h. After that, colonies were counted in the same manner as described above.

### *In vivo* Mono- and Co-cultures of *S. epidermidis* and *E. faecalis*

New Zealand white rabbits (4–5 kg; Hyochang Science, Korea) were used to establish *in vivo* mono- and co-culture models of *S. epidermidis* and *E. faecalis* infection. The rabbits were anesthetized by intramuscular injection of tiletamine-zolazepam (Virbac Korea, France) and xylazine (Bayer, Germany). Each group consisted of five rabbits, and only the right eye was subjected to the experiment.

**Figure 1:**
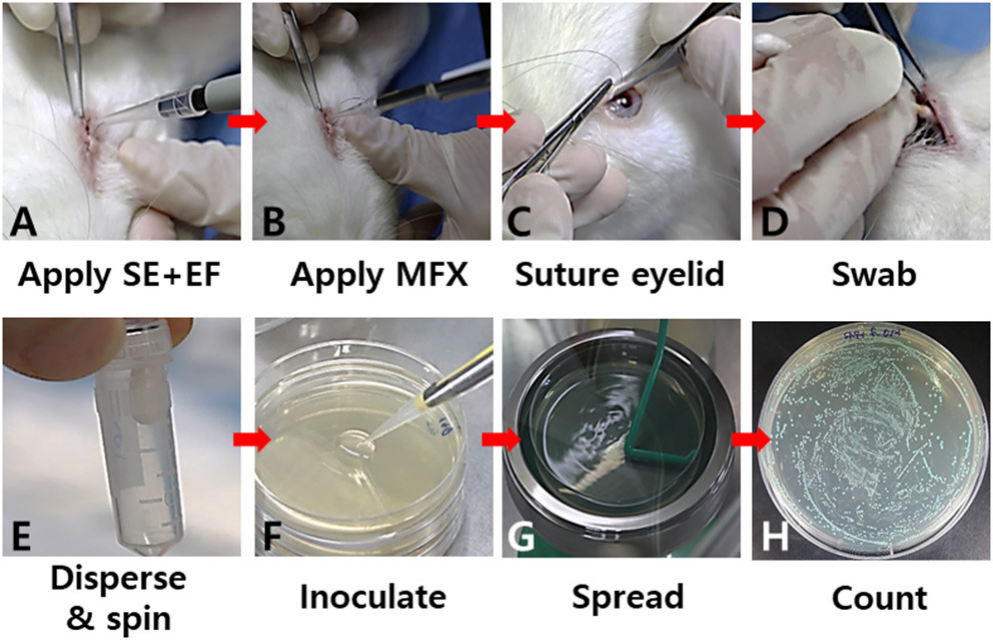
Key steps in the *in vivo* co-culture model of *S. epidermidis* (SE) and *E. faecalis* (EF). A prepared bacterial suspension **(A)** or a topical antibiotic (moxifloxacin; MFX) **(B)** was applied to the lower conjunctival sac. To avoid loss of the bacterial suspension, tarsorrhaphy was performed **(C)**. The bacteria in the inferior cul-de-sac were collected with a sterile cotton-tipped swab **(D)**. The bacterial cells on the swabs were immediately dispersed into a medium in an Eppendorf tube **(E)**. Serial dilutions of the bacterial suspension were seeded on a CDA plate for differential colony counting **(F,G)**. After overnight incubation at 37°C, the numbers of colonies were determined **(H)**.

Either a prepared bacterial suspension or topical antibiotic solution was inoculated into the lower conjunctival sac (Figures 1A,B). To avoid a loss of the solution, tarsorrhaphy was performed using continuous 6–0 nylon (Ethicon, USA) (Figure 1C). To compare the numbers of bacterial cells among the different treatment groups, the bacteria in the inferior cul-de-sac were collected with a sterile, cotton-tipped swab at different time points (4–24 h) after the inoculation (Figure 1D). The swabs were immediately soaked in 1 ml of normal saline in an Eppendorf tube and shaken at 2,800 rpm for 60 s in a microtube homogenizer (BeadBug, Benchmark Scientific, USA) to extract the bacterial cells from the swabs (Figure 1E). Next, the suspension was serially diluted and plated on CDA for differential colony counting (Figures 1F,G). After an overnight incubation at 37°C, the number of *E. faecalis* and *S. epidermidis* colonies were determined as described above (Figure 1H). The study protocol was approved by the Institutional Animal Care and Use Committee of Kosin University College of Medicine (KMAP-18-19). All experiments involving animal subjects were conducted in accordance with the guidelines and regulations of the Association for Research in Vision and Ophthalmology.

For *in vivo* mono-cultures of *S. epidermidis* or *E. faecalis*, 25 μl of the prepared bacterial suspension (10^9^ CFU/ml) was applied to the lower conjunctival sac. For the *in vivo* co-culture model of *S. epidermidis* and *E. faecalis*, various conditions with different ratios of the two bacteria [1:1, 8:2, or 9:1 (*S. epidermidis*: *E. faecalis*)], volumes (50, 25, or 12.5 μl), and concentrations (1 × 10^9^, 2 × 10^9^, or 4 × 10^9^ CFU/ml) were applied. Then, the bacteria were collected at 4, 8, and 16 h after the administration as described above.

### Evaluation of the Effects of Topical Moxifloxacin Administration on the *in vivo* Co-culture Model of *S. epidermidis* and *E. faecalis*

To this end, 9 μl of a *S. epidermidis* suspension (4 × 10^9^ CFU/ml) and 1 μl of an *E. faecalis* suspension (4 × 10^9^ CFU/ml) were employed. Moxifloxacin (5 μl at 0.5% concentration; Tokyo Chemical Industry Co., Japan) was applied to the lower conjunctival sac once immediately after the inoculation of the bacterial suspension or twice (additional treatment 1 h later). The number of bacterial cells in the lower conjunctiva was determined 4 h after the inoculation of the bacterial solution.

### Statistics

All *in vitro* experiments were conducted three times independently and animal experiments were conducted at least three to five times with a group of five animals. Paired Student's *t*-test was performed to assess the significance of differences between two groups. All statistical analyses were performed using GraphPad Prism (version 6.03). Statistical significance was defined as a *p*-value < 0.05.

## Results

### Growth Curves of *in vitro* Mono-Cultures and Co-cultures of *S. epidermidis* and *E. faecalis*

**Figure 2:**
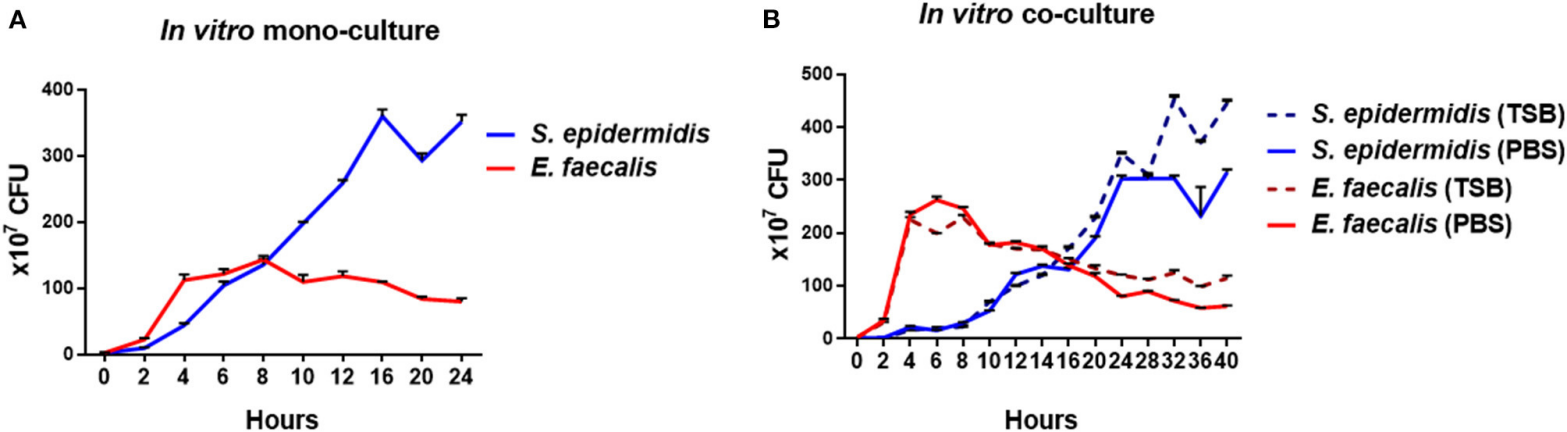
Growth curves of *in vitro* mono-cultures and co-cultures of *S. epidermidis* and *E. faecalis*. For the mono-cultures, *S. epidermidis* and *E. faecalis* were cultured separately in TSB, and the numbers of bacterial cells were determined by quantifying CFUs every 2-4 h **(A)**. For the co-cultures, the two bacterial species were mixed at the ratio of 1:1 and cocultivated in either TSB or PBS. The CFUs on the CDA plates were counted every 2-4 h **(B)**.

When *S. epidermidis* was cultivated alone in TSB, its growth peaked and plateaued at approximately 16 h (Figure 2A). When *E. faecalis* was cultured alone in TSB, its growth peaked and plateaued at ~6 h, which was faster than that of *S. epidermidis*. When *S. epidermidis* and *E. faecalis* were cocultivated, peak growth of *S. epidermidis* got delayed to approximately 24 h (Figure 2B). In addition, the concentration of *E. faecalis* cells was 1.86-fold higher in the co-culture with *S. epidermidis* than that in mono-culture.

### Growth Patterns of *S. epidermidis* and *E. faecalis* Cultivated With Various Concentrations of a Culture Supernatant

**Figure 3:**
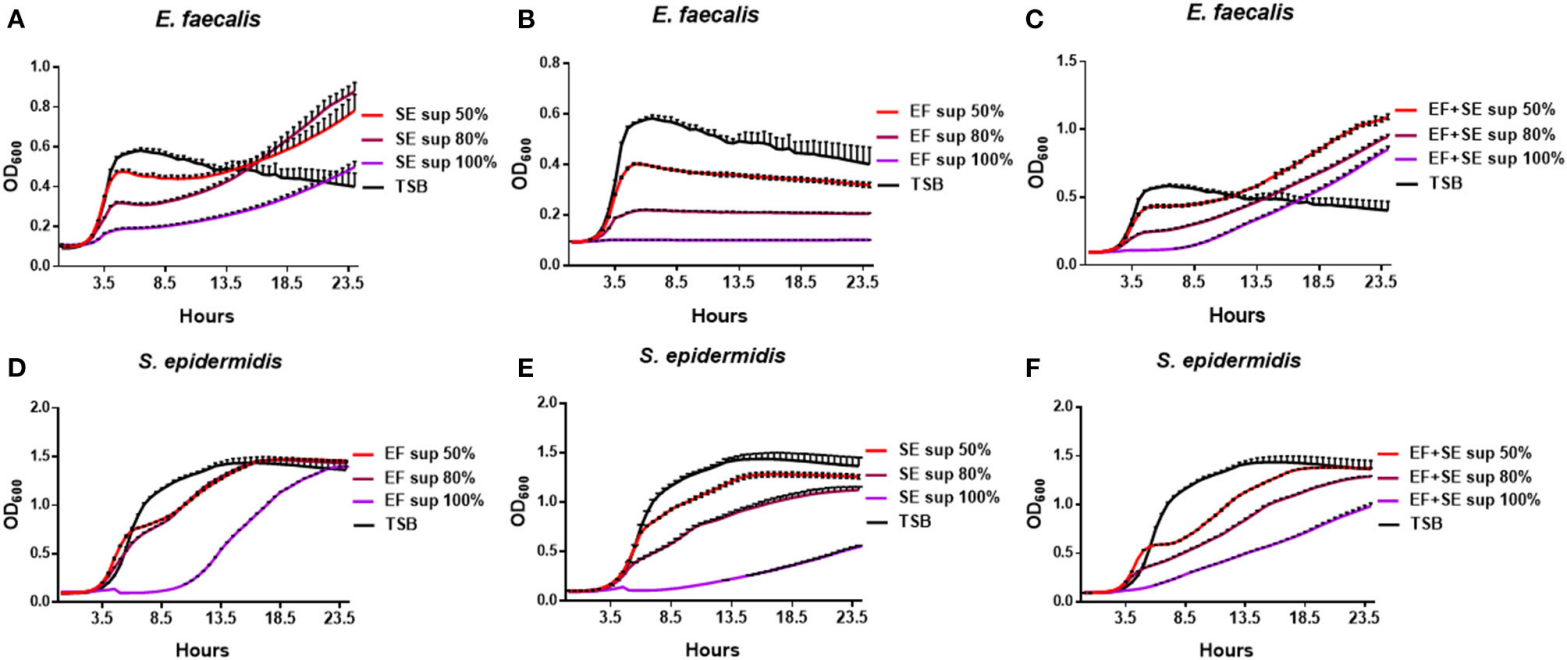
Growth patterns of *S. epidermidis* (SE) and *E. faecalis* (EF) cultivated with various concentrations of a culture supernatant (sup). *E. faecalis* was cultured with media containing a supernatant (0, 50, 80, or 100%) of *S. epidermidis* mono-culture **(A)**, of *E. faecalis* mono-culture **(B)**, or of the *S. epidermidis* + *E. faecalis* co-culture **(C)** and the growth rates were measured every 30 min. In the same way, *S. epidermidis* was cultivated with a supernatant of *E. faecalis* mono-culture **(D)**, *S. epidermidis* mono-culture **(E)**, or of the co-culture **(F)** and growth curves were constructed.

To further analyze the difference in the growth patterns between the mono-cultures and co-cultures of *S. epidermidis* and *E. faecalis*, these microbes were cultivated alone with various concentrations of a culture supernatant from *S. epidermidis* or *E. faecalis* mono-cultures or co-cultures (Figure 3). When *E. faecalis* was cultured with a supernatant from mono-cultures or co-culture of *S. epidermidis* or *E. faecalis*, the proliferation rate decreased in a supernatant concentration-dependent manner (Figures 3A–C, Supplementary Figures 1A-C). In the case of *S. epidermidis*, when cultivated with a *S. epidermidis, E. faecalis*, or co-culture supernatant, the initial growth rates (first 4 h) were similar to that of the control except for samples with the 100% supernatant. In contrast, the growth rate of a later phase slowed down, taking a long time to reach the maximum cell concentration (Figures 3D–F, Supplementary Figures 1D-F). Notably, when *E. faecalis* was cultured with a supernatant from *S. epidermidis* mono-culture or co-culture, the growth slowdown and the delay of maximum cell concentration shown in Figure 3B were overcome: we noticed a slow increase of cell proliferation over time (Figures 3A,C). In other words, although the maximum cell concentration decreased as the concentration of the supernatant increased, the growth of *E. faecalis* cultivated with media containing the supernatant of *S. epidermidis* was found to recover and reached the final OD_600_ higher than that of the mono-culture in TSB. These results suggested that the *S. epidermidis* culture contains some substances that induce the increase in the cell concentration of *E. faecalis*, which may explain the significant increase of maximum cell concentration of *E. faecalis* in the co-culture with *S. epidermidis*.

### Effects of Moxifloxacin on *in vitro* Co-cultures of *S. epidermidis* and *E. faecalis*

**Figure 4:**
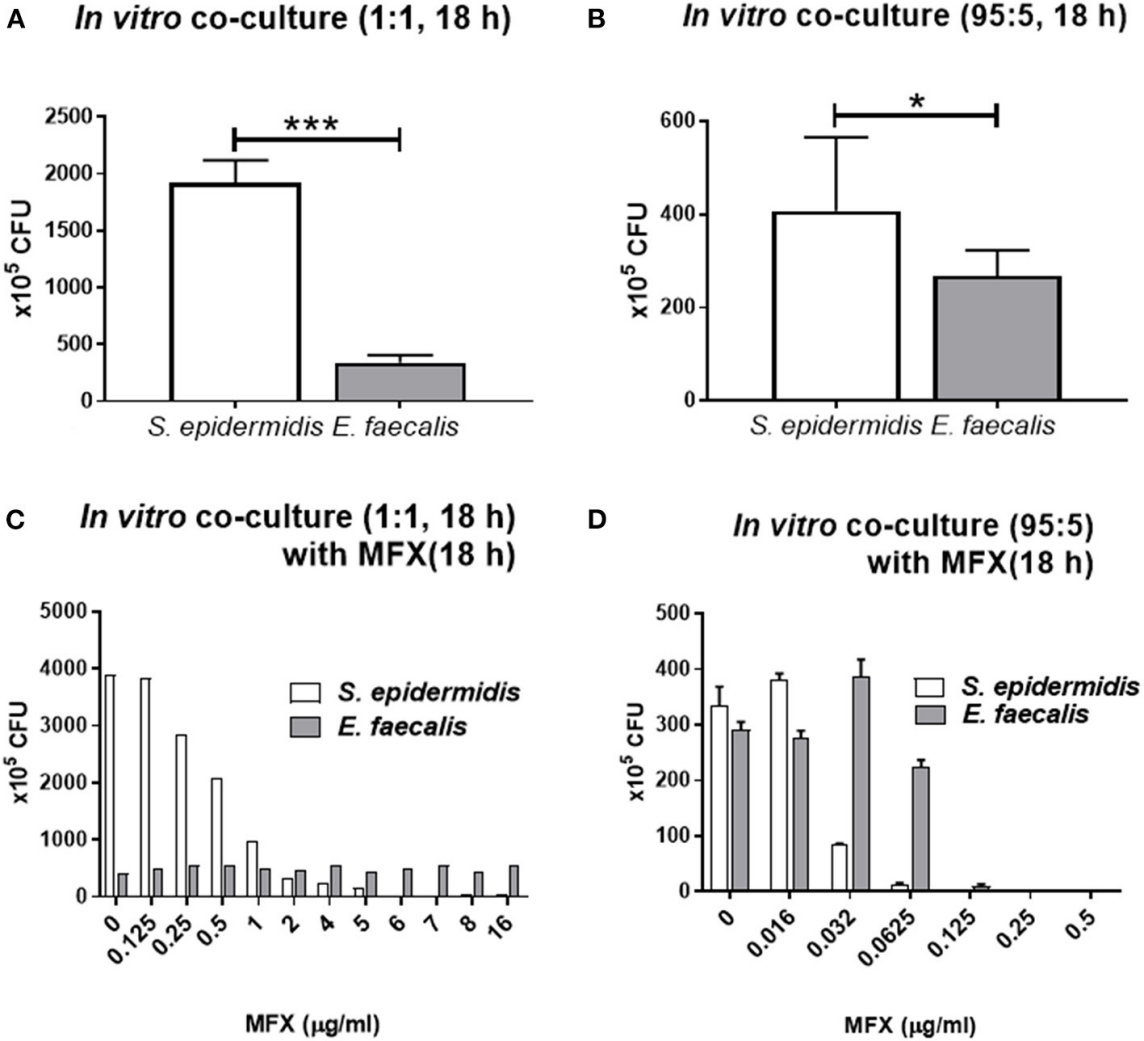
*In vitro* co-culture modeling of *S. epidermidis* (SE) and *E. faecalis* (EF) and their responses of bacteria to moxifloxacin treatment. *S. epidermidis* and *E. faecalis* were mixed at the ratio of 1:1 and cocultured for 18 h, and their CFUs were quantified **(A)**. Similarly, a mixture in the 95:5 ratio (SE:EF) was cultivated for 18 h **(B)**. For the co-culture with moxifloxacin (MFX) treatment, the bacterial mixture in the 1:1 ratio was cocultured for 18 h and then treated with various concentrations of moxifloxacin. The CFUs were counted after 14 h moxifloxacin treatment **(C)**. When *S. epidermidis* and *E. faecalis* were mixed at the ratio of 95:5, the moxifloxacin treatment was started immediately, without the 18 h culturing period, and the CFUs were counted after 18 h moxifloxacin treatment **(D)**. Means and S.D. (*n* = 3) are shown as columns and error bars, respectively. Asterisks at the top of bars denote statistically significant differences (*, *p* < 0.05; ***, *p* < 0.001; student *t*-test).

When *S. epidermidis* and *E. faecalis* were cocultivated at the ratio of 1:1 or 95:5 for 18 h, the final cellular concentration of the two species was different (Figures 4A,B). The concentrations of *S. epidermidis* and *E. faecalis* cells were 1,925 ± 195 × 10^5^ and 338 ± 68 × 10^5^ CFU /ml, respectively, when cocultured at 1:1 (*p* < 0.0001, Figure 4A) and 408 ± 158 x 10^5^ and 268 ± 55.0 x 10^5^, respectively, when cocultured at 95:5 (*p* = 0.028, Figure 4B). This difference was similar to the result shown in Figure 2B, in which the numbers of colonies of the cocultured bacteria, mixed in the ratio of 1:1, are compared over time. In the co-culture, the *E. faecalis* count initially increased sharply at 4–6 h and then decreased, while the growth of *S. epidermidis* was initially delayed and then was good between 12 and 20 h. For this reason, the 1:1 co-culture had a significantly smaller number of *E. faecalis* cells than *S. epidermidis* cells after 18 h. Of note, the numbers of *S. epidermidis* colonies were markedly different between the 1:1 and 95:5 co-cultures, whereas the numbers of *E. faecalis* colonies were similar.

When 1:1 co-cultures were treated with moxifloxacin at various concentrations for 18 h, the numbers of *S. epidermidis* cells rapidly decreased at 2 μg/ml moxifloxacin, while the numbers of *E. faecalis* cells, did not significantly change even at a moxifloxacin concentration of 16 μg/ml (Figure 4C). On the contrary, when *S. epidermidis* and *E. faecalis* were mixed at 95:5 and immediately treated with moxifloxacin for 18 h (without co-cultivation for 18 h beforehand), the numbers of *S. epidermidis* colonies stared to decline at 0.032 μg/ml moxifloxacin, and no *S. epidermidis* colonies were detectable at 0.125 μg/ml. The decrease of *E. faecalis* colonies were observed at 0.125 μg/ml and no colonies at 0.25 μg/ml (Figure 4D). Therefore, when the two bacteria were cocultured for 18 h before the exposure to moxifloxacin, the resistance to this drug strengthened for bacteria. In particular, *E. faecalis* co-cultured with *S. epidermidis* survived with moxifloxacin above 16 μg/ml.

### *In vivo* Co-culture Modeling With Different Volumes and Ratios of *S. epidermidis* and *E. faecalis* Cell Suspensions

**Figure 5:**
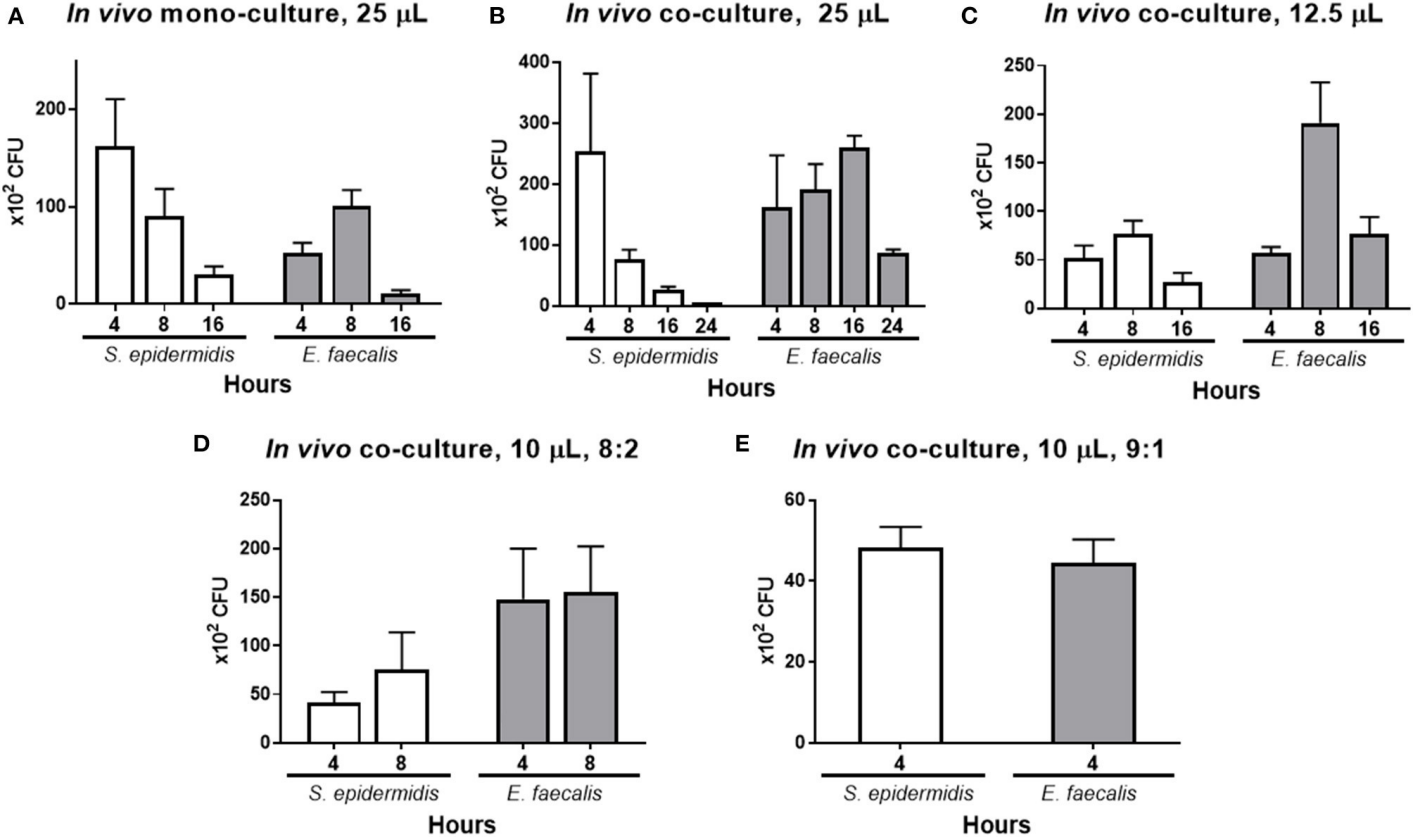
*In vivo* co-culture modeling and effects of the mixing ratio and volume of the numbers of *S. epidermidis* (SE) and *E. faecalis* (EF) cells. As a control, the mono-cultures of each bacterium were inoculated (25 μl of a 1 × 10^9^ CFU/ml suspension) into the lower conjunctival sac of a rabbit, and the bacteria CFUs were quantified at 4, 8, and 16 h post-inoculation **(A)**. Under the same conditions, co-culture inoculum was prepared by mixing the two bacteria at the 1:1 ratio (25 μl with total volume, with a total bacterial concentration of 1 × 10^9^ CFU/ml). After inoculation, the CFUs were counted at 4, 8, 16, and 24 h **(B)**. Half volume with a double-concentrated suspension was prepared by reducing the inoculum volume to 12.5 μl and raising the cell concentration to 2 × 10^9^ CFU/ml. After inoculation, the CFUs were counted at 4, 8, and 16 h **(C)**. Next, the total inoculum volume was reduced to 10 μl at a concentration of 4 × 10^9^ CFU/ml, and different ratios of *S. epidermidis* to *E. faecalis* were tested. First, the microbes in the ratio of 8:2 (SE:EF) were inoculated, and the CFUs were counted at 4 and 8 h after the inoculation **(D)**. Finally, the bacteria in the ratio of 9:1 (SE:EF) were inoculated and the CFU were counted at 4 h after inoculation **(E)**, and this ratio was chosen for the test of moxifloxacin treatment. Means and S.D. (*n* = 3) are shown as columns and error bars, respectively.

In the *in vivo* mono-cultures (25 μl) with the inoculum concentration of 10^9^ CFU/ml, the number of *S. epidermidis* colonies gradually diminished at every time point, whereas the number of *E. faecalis* colonies increased until 8 h and then decreased (Figure 5A). When these bacteria were co-cultured, the number of *S. epidermidis* colonies decreased at every time point and was close to zero at 24 h, whereas the number of *E. faecalis* increased until 16 h and then decreased (Figure 5B). In comparison to *in vivo* mono-culture, *E. faecalis* had higher number of cells for longer time in the *in vivo* co-culture. When the inoculum concentration was doubled to 2 × 10^9^ CFU/ml by reducing the inoculum volume in half from 25 to 12.5 μl, the number of *S. epidermidis* colonies were low at 4 h, slightly increased at 8 h and then declined, which differed from the result above, and the *E. faecalis* count went up until 8 h and then decreased (Figure 5C).

In order to implement the antibiotic treatment, the volume of the suspension of inoculated bacteria was reduced further, and the ratio of the bacteria cells was adjusted. The total volume of the bacterial inoculum was reduced to 10 μl (4 × 10^9^ CFU/ml), and the ratios of *S. epidermidis* and *E. faecalis* cells were 8:2 and 9:1 (Figures 5D,E). When the ratio was 8:2, the *S. epidermidis* count increased until 8 h, whereas the *E. faecalis* count was stable for 8 h. When the initial ratio was 9:1, the numbers of *S. epidermidis* and *E. faecalis* cells were almost identical at 4 h.

### Survival of *in vivo* Co-cultures After Moxifloxacin Treatment

**Figure 6:**
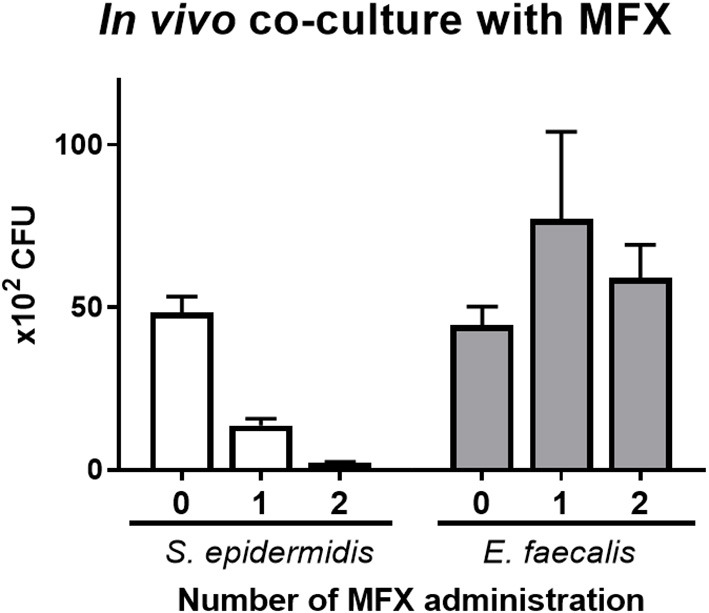
The influence of moxifloxacin on the *in vivo* co-culture model of *S. epidermidis* (SE) and *E. faecalis* (EF). A bacterial suspension was prepared in the ratio of 9:1 (SE:EF) in a total volume of 10 μl (4 × 10^9^ CFU/ml) and was inoculated into a lower conjunctival sac of rabbits. A moxifloxacin solution (5 μl at a 0.5% concentration) was administered zero, one, or two times. The first treatment with moxifloxacin was administered immediately after the bacterial suspension inoculation, and the second treatment was performed 1 h later. The CFUs were counted at 4 h after bacterial inoculation. Means and S.D. (*n* = 3) are shown as bars and error bars, respectively.

The effects of moxifloxacin eye drops were tested on the *in vivo* co-culture model of *S. epidermidis* and *E. faecalis*. These microbes (4 × 10^9^ CFU/ml each) were inoculated (9 and 1 μl, respectively), and moxifloxacin was administered zero, one, or two times. The numbers of *S. epidermidis* and *E. faecalis* colonies were determined at 4 h post inoculation (Figure 6). The number of *S. epidermidis* colonies diminished significantly as the number of moxifloxacin treatment increased. In contrast, the number of *E. faecalis* colonies remained constant, regardless of the moxifloxacin administration scheme. Moreover, when moxifloxacin was administered once, the number of *E. faecalis* colonies tended to go up.

## Discussion

In this study, we developed *in vitro* and *in vivo* co-culture models of *S. epidermidis* and *E. faecalis* to evaluate the impact of moxifloxacin on the survival of these two bacteria. *E. faecalis* has significantly higher resistance to moxifloxacin than *S. epidermidis* does, and administration of moxifloxacin likely allows for selective survival of *E. faecalis* on the ocular surface. The *in vitro* and *in vivo* co-culture models clearly showed that moxifloxacin treatment reduce the number of *S. epidermidis* but not the number of *E. faecalis*. These suggest that if there is a small number of *E. faecalis* cells in the conjunctiva, then administration of moxifloxacin promotes not only the selective survival of *E. faecalis* but also its domination in conjunctival sac microflora.

Recently, administration of prophylactic antibiotics before and after intraocular surgery became a common practice, even though there is no clear evidence that it prevents infection. If fluoroquinolone is utilized as a prophylactic antibiotic perioperatively, it is expected to be effective against *S. epidermidis* present on the ocular surface but not against *E. faecalis*, hence changing conjunctiva microflora. The changed conjunctiva microflora provides more opportunities for *E. faecalis* to enter the intraocular space through the surgical wounds, resulting PE.

In our study, *in vitro* co-culture of *S. epidermidis* and *E. faecalis* increased the maximum cell concentration of *E. faecalis* in contrast to the mono-cultures. The different growth patterns between mono- and co-cultures is likely related to direct co-culture of the two bacteria. In other words, it is highly likely that the proliferation of *E. faecalis* is induced in the presence of *S. epidermidis*. To explain this difference, the bacteria were cultivated with various concentrations of a culture supernatant from each bacterial species. The experiments using culture supernatants have many limitations to represent the direct co-culture condition. For example, using the culture supernatant of stationary phase may exclude some components of exponential phase, and the nutrient-reduced culture supernatant may hinder normal growth of bacteria. The supernatant of stationary phase rather than exponential phase was chosen because we thought that bacterial growth stage in the *in vivo* condition, where bacteria inhabit on the ocular surface, may be closer to the stationary phase than exponential phase. When the supernatant of stationary phase is used, the effect of nutrient reduction can be an issue. When 0, 50, 80, 100% of supernatant with culture media TSB was used, the effect of nutrient-reduced supernatant to the bacterial growth was shown (Figure 3). However, the purpose of this experiment is to observe the changes in the growth pattern of bacteria affected by bacterial culture supernatant. For this purpose, we were able to observe that the supernatant of *S. epidermidis* culture induced the increase of the cell density of *E. faecalis* above their maximum cell concentration in the standard culture.

The growth patterns shown in the *in vitro* co-culture model were also observed in the *in vivo* co-culture model. When *E. faecalis* and *S. epidermidis* were administered to the lower conjunctival sac of rabbits, the number of *S. epidermidis* cells decreased with time, whereas the number of *E. faecalis* cells increased for up to 16 h. These data suggest that if *E. faecalis* enters the conjunctival sac before or after intraocular surgery, it is likely to proliferate in the conjunctival sac for at least 16 h. Therefore, great care should be taken not to contaminate the ocular surface with the bacteria before and after intraocular surgery.

We next sought to determine whether there is a change in antibiotic sensitivity when the two bacteria were co-cultured. When the bacteria were mixed (at a ratio of 95:5) and immediately treated with moxifloxacin, without a co-culturing period, no *S. epidermidis* colonies were detectable in the presence of 0.125 μg/ml moxifloxacin, and there were no *E. faecalis* colonies in the presence of 0.25 μg/ml moxifloxacin. Of note, when *S. epidermidis* and *E. faecalis* were mixed (at a ratio of 1:1), co-cultured for 14 h and only then treated with moxifloxacin, the antibiotic concentration effective against *S. epidermidis* was much higher, 6 μg/ml and, moreover, *E. faecalis* became resistant even to 16 μg/ml moxifloxacin. This phenomenon was observed in the *in vivo* co-culture model too. In the *in vivo* model, when 0.5% moxifloxacin was administered twice with an 1 h interval, *S. epidermidis* almost disappeared, but *E. faecalis* persisted. These findings also imply that not only in the conjunctival sac but also in the cases of endophthalmitis, where the bacteria co-proliferate in the vitreous cavity, *E. faecalis* may become more resistant to fluoroquinolone antibiotics.

Fluoroquinolone eye drops are widely used before and after intraocular surgery to prevent PE. It is still debatable whether these antibiotics reduces the risk of infectious endophthalmitis. Although these antibiotics are very likely to effectively eliminate CNS, including *S. epidermidis*, in the conjunctival microflora, our data suggest that moxifloxacin may have little antimicrobial activity toward the *Enterococcus* spp. that are present in the conjunctiva.

Most of the bacteria that cause PE are those already present in the conjunctiva. Because the most prevalent bacteria in the conjunctiva are CNS, including *S. epidermidis*, they have been the major causative agent of PE after surgery. On the other hand, several recent reports on PE in Korea, Sweden, and Taiwan indicated that *E. faecalis* has become the top causative agent of PE (6, 9–11). *Enterococcus* spp. have been relatively rare but are of special clinical importance because they cause a fulminant and destructive disease course, and poor visual outcomes of infected patients (7, 25–27). In one study, only 17% of patients with *E. faecalis* endophthalmitis achieved a final visual acuity of 20/400 or better (28). Therefore, the emergence of *E. faecalis* as a cause of PE may affect visual outcomes after treatment of PE.

This reshuffling of major causative isolates of PE is especially obvious in a series of nationwide, multicenter, prospective studies in Sweden. In the first report, covering the 1998 national prospective survey, the most common bacteria that caused PE were CNS (15/41, 41.37%) (29). In the second report, covering the 1999–2001 survey, CNS were still the most common isolates at 32.97% (30/91), followed by *Enterococcus* spp. at 25.27% (23/91) (30). In the third report, covering the 2002–2004 survey, CNS were the most common isolates at 39.08% (34/87), again followed by *Enterococcus* spp. at 28.73% (25/87) (31). In the most recent report, covering the 2005–2010 survey, *Enterococcus* spp. were the most common isolates at 39.13% (42/115), followed by CNS at 30.43% (35/115) (10). Looking at the results of these four surveys covering 12 years, we can see that the percentage of PE cases caused by *Enterococcus* spp. gradually increased. Those authors hypothesized that the selection pressure applied by antibiotics has caused the other species to become the leading causative microorganisms of PE (32).

One of key tasks for the researchers in this field is establishing a proper experimental model to investigate the shifts of the major causative isolates of PE. Challenges for the studies on this complex environment of the ocular surface are not limited to the presence of many cell types and bacterial species: there is also substantial variations of the microbiota among individuals. Mimicking such complex ocular surface conditions is difficult in an *in vitro* model. Therefore, creating *in vivo* model is essential for researching these changes of ocular pathological processes. Our proposed *in vivo* animal model may be suitable for studying the shifts of the major causative isolates for understanding PE pathogenesis, and for evaluating novel therapeutics.

In conclusion, we successfully created *in vitro* and *in vivo* co-culture models of *S. epidermidis* and *E. faecalis*. By means of these models, we confirmed that moxifloxacin, a fourth-generation fluoroquinolone, is effective against *S. epidermidis*, but not *E. faecalis*. Our results provide supporting evidences that the administration of fluoroquinolone as perioperative ophthalmic treatment is an important contributing factor of the change in the major causative agent of PE from *S. epidermidis* to *E. faecalis*. The co-culture models of this study can be a useful tool to develop an effective antibiotic that can control PE caused by *E. faecalis*.

## Data Availability Statement

The original contributions presented in the study are included in the article/Supplementary Material, further inquiries can be directed to the corresponding authors.

## Ethics Statement

The animal study was reviewed and approved by the Institutional Animal Care and Use Committee of Kosin University College of Medicine (KMAP-18-19).

## Author Contributions

SL conceived of the project and created all instructional materials. SL and HK prepared all figures and wrote the manuscript. JK and IP performed data analysis and critical review the manuscript. All the authors contributed to the article and approved the submitted version.

## Conflict of Interest

The authors declare that the research was conducted in the absence of any commercial or financial relationships that could be construed as a potential conflict of interest.

## Publisher's Note

All claims expressed in this article are solely those of the authors and do not necessarily represent those of their affiliated organizations, or those of the publisher, the editors and the reviewers. Any product that may be evaluated in this article, or claim that may be made by its manufacturer, is not guaranteed or endorsed by the publisher.

## Supplementary Material

Supplementary Figure S1 Logarithmic growth curves of in vitro mono-cultures and co-cultures of *S. epidermidis* and *E. faecalis*. Growth patterns of *S. epidermidis* (SE) and *E. faecalis* (EF) cultivated with various concentrations of a culture supernatant (sup). *E. faecalis* was cultured with media containing a supernatant (0, 50%, 80%, or 100%) of *S. epidermidis* mono-culture **(A)**, of *E. faecalis* mono-culture **(B)**, or of the *S. epidermidis* + *E. faecalis* co-culture **(C)** and the growth rates were measured every 30 min. In the same way, *S. epidermidis* was cultivated with a supernatant of *E. faecalis* mono-culture **(D)**, *S. epidermidis* mono-culture **(E)**, or of the co-culture **(F)** and growth curves were constructed.

## References

[1] Weikert MP. Update on bimanual microincisional cataract surgery. Curr Opin Ophthalmol. (2006) 17:62–7. 10.1097/01.icu.0000193069.32369.e1 16436926

[2] Yorston D. Cataract complications. Community Eye Heal J. (2008) 21:1–3. PMC2377378 18504465

[3] Wong TY Chee S-P. The epidemiology of acute endophthalmitis after cataract surgery in an Asian population. Ophthalmology. (2004) 111:699–705. 10.1016/j.ophtha.2003.07.014 15051201

[4] Greenberg PB Tseng VL Wu W-C Liu J Jiang L Chen CK. Prevalence and predictors of ocular complications associated with cataract surgery in United States veterans. Ophthalmology. (2011) 118:507–14. 10.1016/j.ophtha.2010.07.023 21035868

[5] Nam KY Lee JE Lee JE Jeung WJ Park JM Park JM. Clinical features of infectious endophthalmitis in South Korea: a five-year multicenter study. BMC Infect Dis. (2015) 15:177. 10.1186/s12879-015-0900-5 25885441 PMC4399575

[6] Kim HW Kim SY Chung IY Lee JE Lee JE Park JM. Emergence of *Enterococcus* species in the infectious microorganisms cultured from patients with endophthalmitis in South Korea. Infection. (2014) 42:113–8. 10.1007/s15010-013-0530-z 24072645

[7] Han DP Vine AK Blodi BA Elner SG Johnson MW Khanderia S. Microbiologic factors and visual outcome in the endophthalmitis vitrectomy study. Am J Ophthalmol. (1996) 122:830–46. 10.1016/S0002-9394(14)71959-2 8956638

[8] Bannerman TL Rhoden DL McAllister SK Miller JM Wilson LA. The source of coagulase-negative *Staphylococci* in the Endophthalmitis vitrectomy study. Arch Ophthalmol. (1997) 115:357. 10.1001/archopht.1997.01100150359008 9076208

[9] Kim JK Nam KY Chung IY Jeung WJ Kwon YH Park JM. Emerging *Enterococcus* isolates in postoperative endophthalmitis by selection pressure of fluoroquinolones: an 11-year multicenter and experimental study. Emerg Microbes Infect. (2020) 9:1892–9. 10.1080/22221751.2020.1810134 32811346 PMC7473211

[10] Friling E Lundström M Stenevi U Montan P. Six-year incidence of endophthalmitis after cataract surgery: Swedish national study. J Cataract Refract Surg. (2013) 39:15–21. 10.1016/j.jcrs.2012.10.037 23245359

[11] Teng YT Teng MC Kuo HK Fang PC Wu PC Chen CH. Isolates and antibiotic susceptibilities of endophthalmitis in postcataract surgery: a 12-year review of culture-proven cases. Int Ophthalmol. (2017) 37:513–8. 10.1007/s10792-016-0288-2 27422143

[12] Mountcastle SE Cox SC Sammons RL Jabbari S Shelton RM Kuehne SA. A review of co-culture models to study the oral microenvironment and disease. J Oral Microbiol. (2020) 12:1773122. 10.1080/20002297.2020.1773122 32922679 PMC7448840

[13] Iskeleli G Bahar H Eroglu E Torun MM Ozkan S. Microbial changes in conjunctival flora with 30-day continuous-wear silicone hydrogel contact lenses. Eye Contact Lens. (2005) 31:124–6. 10.1097/01.ICL.0000141923.63458.DF 15894879

[14] Yamauchi Y Minoda H Yokoi K Maruyama K Kumakura S Usui M. Conjunctival flora in patients with human immunodeficiency virus infection. Ocul Immunol Inflamm. 13:301–4. 10.1080/09273940590951106 16159721

[15] Kusbeci T Kusbeci OY Aktepe OC Yavas G Ermis SS. Conjunctival flora in patients with Parkinson's disease. Curr Eye Res. (2009) 34:251–6. 10.1080/02713680902725970 19373572

[16] Adam M Balci M Bayhan HA Inkaya AÇ Uyar M Gürdal C. Conjunctival flora in diabetic and nondiabetic individuals. Turkish J Ophthalmol. (2015) 45:193–6. 10.4274/tjo.33230 27800231 PMC5082240

[17] Hwang DG. Fluoroquinolone resistance in ophthalmology and the potential role for newer ophthalmic fluoroquinolones. Surv Ophthalmol. (2004) 49:S79–83. 10.1016/j.survophthal.2004.01.004 15028483

[18] Chang DF Braga-Mele R Mamalis N Masket S Miller KM Nichamin LD. Prophylaxis of postoperative endophthalmitis after cataract surgery: results of the 2007 ASCRS member survey. J Cataract Refract Surg. (2007) 33:1801–5. 10.1016/j.jcrs.2007.07.009 17889779

[19] Liesegang TJ. Perioperative antibiotic prophylaxis in cataract surgery. Cornea. (1999) 18:383–402. 10.1097/00003226-199907000-00001 10422849

[20] Agardh C-DD Agardh E Torffvit O Agardh C-DD Torffvit O Agardh E. The prognostic value of albuminuria for the development of cardiovascular disease and retinopathy: a 5-year follow-up of 451 patients with type 2 diabetes mellitus. Diabetes Res Clin Pract. (1996) 32:35–44. 10.1016/0168-8227(96)01218-1 8803480

[21] Adhi M Alwassia AA Duker JS. Analysis of choroidal thickness in eyes treated with focal laser photocoagulation using SD-OCT. Can J Ophthalmol. (2013) 48:535–8. 10.1016/j.jcjo.2013.05.010 24314418

[22] Aderman CM Chao DL Obeid A Schwartz DM Bhisitkul RB Chiu CS. Bilateral *Enterococcus faecalis* endophthalmitis with multiple recurrences. Retin Cases Brief Rep. (2021) 15:38–42. 10.1097/ICB.0000000000000722 29489562

[23] Abbasi J. A retinal scan for Alzheimer disease. JAMA. (2017) 318:1314. 10.1001/jama.2017.15192 29049570

[24] Dave SB Toma HS Kim SJ Toma HS Kim SJ Dave SB. Changes in ocular flora in eyes exposed to ophthalmic antibiotics. Ophthalmology. (2013) 120:937–41. 10.1016/j.ophtha.2012.11.005 23415422

[25] Benz MS Scott IU Flynn HW Unonius N Miller D Flynn Jr HW. Endophthalmitis isolates and antibiotic sensitivities: a 6-year review of culture-proven cases. Am J Ophthalmol. (2004) 137:38–42. 10.1016/S0002-9394(03)00896-1 14700642

[26] Choi S Hahn TW Osterhout G O'Brien TP. Comparative intravitreal antibiotic therapy for experimental *Enterococcus faecalis* endophthalmitis. Arch Ophthalmol. (1996) 114:61. 10.1001/archopht.1996.01100130057009 8540852

[27] Chen K-J Lai C-C Sun M-H Chen T-L Yang K-J Kuo Y-H. Postcataract endophthalmitis caused by *Enterococcus faecalis*. Ocul Immunol Inflamm. (2009) 17:364–9. 10.3109/09273940903105110 19831574

[28] Scott IU Loo RH Flynn HW Miller D. Endophthalmitis caused by *Enterococcus faecalis*: antibiotic selection and treatment outcomes. Ophthalmology. (2003) 110:1573–7. 10.1016/S0161-6420(03)00502-5 12917175

[29] Montan P Lundström M Stenevi U Thorburn W. Endophthalmitis following cataract surgery in Sweden.The 1998 national prospective survey. Acta Ophthalmol Scand. (2002) 80:258–61. 10.1034/j.1600-0420.2002.800305.x 12059862

[30] Wejde G Montan P Lundström M Stenevi U Thorburn W. Endophthalmitis following cataract surgery in Sweden: national prospective survey 1999-2001. Acta Ophthalmol Scand. (2005) 83:7–10. 10.1111/j.1600-0420.2005.00377.x 15715550

[31] Wejde G Stenevi U Lundström M Thorburn W Montan P Lundström M. Endophthalmitis after cataract surgery. Ophthalmology. (2007) 114:866–70. 10.1016/j.ophtha.2006.11.025 17324467

[32] Friling E Montan P. Bacteriology and cefuroxime resistance in endophthalmitis following cataract surgery before and after the introduction of prophylactic intracameral cefuroxime: a retrospective single-centre study. J Hosp Infect. (2019) 101:88–92. 10.1016/j.jhin.2018.02.005 29432821

