# Supplementary figures and images for: Establishing in vitro and in vivo Co-culture Models of Staphylococcus epidermidis and Enterococcus faecalis to Evaluate the Effect of Topical Fluoroquinolone on Ocular Microbes
Source: Front Med (Lausanne). 2021 Dec 20;8:670199. doi: 10.3389/fmed.2021.670199 (PMC8720975; doi:10.3389/fmed.2021.670199)

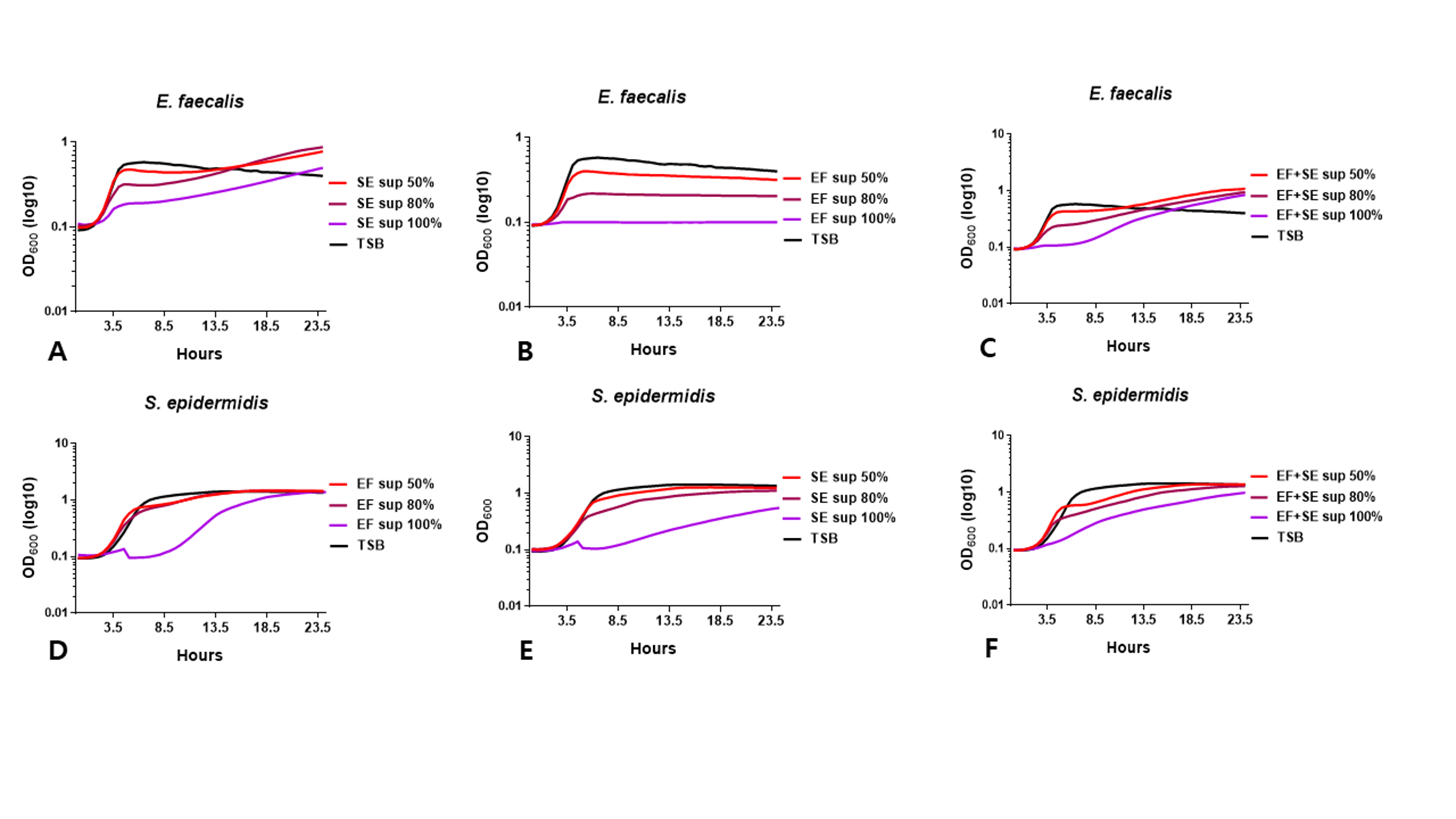

Supplement: Supplementary Figure S1 — Logarithmic growth curves of in vitro mono-cultures and co-cultures of S. epidermidis and E. faecalis. Growth patterns of S. epidermidis (SE) and E. faecalis (EF) cultivated with various concentrations of a culture supernatant (sup). E. faecalis was cultured with media containing a supernatant (0, 50%, 80%, or 100%) of S. epidermidis mono-culture (A), of E. faecalis mono-culture (B), or of the S. epidermidis + E. faecalis co-culture (C) and the growth rates were measured every 30 min. In the same way, S. epidermidis was cultivated with a supernatant of E. faecalis mono-culture (D), S. epidermidis mono-culture (E), or of the co-culture (F) and growth curves were constructed. [file Image_1.TIF]
